# Unmet needs of biochemical biomarkers for human prion diseases

**DOI:** 10.1080/19336896.2024.2349017

**Published:** 2024-05-12

**Authors:** Peter Hermann, Inga Zerr

**Affiliations:** a Department of Neurology, University Medical Center Göttingen, Göttingen, Germany; b German Center for Neurodegenerative Diseases, Göttingen, Germany

**Keywords:** Biomarkers, cerebrospinal fluid biomarkers, Creutzfeldt-Jakob disease, diagnosis, disease onset, prion diseases

## Abstract

Although the development of aggregation assays has noticeably improved the accuracy of the clinical diagnosis of prion diseases, research on biomarkers remains vital. The major challenges to overcome are non-invasive sampling and the exploration of new biomarkers that may predict the onset or reflect disease progression. This will become extremely important in the near future, when new therapeutics are clinically evaluated and eventually become available for treatment. This article aims to provide an overview of the achievements of biomarker research in human prion diseases, addresses unmet needs in the field, and points out future perspectives.

## Developments in clinical diagnostic tests for human prion diseases

Over the past three decades, significant progress has been made in the area of diagnostic tests available to support the clinical diagnosis of human prion diseases [1]. In the 1970s and 1980s of the last century, clinician faced the problem that the diagnosis of a prion disease could be confirmed only by means of post-mortem brain autopsy or biopsy, which was the gold standard to diagnose the disease at that time. Since then, the field has significantly moved forward. Systematic studies on clinical Creutzfeldt-Jakob Disease (CJD) [2] cases revealed periodic sharp- and slow-wave complexes in electroencephalogram (EEG) as a typical feature that appears in the middle and late disease stages. Almost a decade later, the focus of searching for a specific disease-related biomarker shifted to the cerebrospinal fluid (CSF). It has been known for many years that CSF in CJD is not remarkably changed, and no signs of a typical inflammatory response are seen. However, proteomic analyses revealed the presence of two proteins, called p 130/131 [3,4], which were detectable only in CJD and herpes simplex encephalitis. Subsequently, these two proteins were identified as members of the 14-3-3 family [5]. This was a great advantage since antibodies against 14-3-3 protein were viable, and the CSF could be tested for the presence of those proteins by western blotting (and later ELISA). Since then, many studies have confirmed that 14-3-3 proteins are detectable in the CSF of most patients with sporadic CJD. Although the specificity of these proteins has been questioned, most studies reported correct negative results in 80–96% of controls [6,7], depending on the cohort analysed. Another biomarker that displays similar characteristics is the tau protein [8]. At the turn of the century, magnetic resonance imaging (MRI) became an important clinical test. High signal abnormalities in the basal ganglia, thalamus, and cortical areas on magnetic resonance FLAIR and diffusion-weighted images (DWI) are characteristic of prion diseases [9,10]. Some of them may even be detected preclinically [11–15]. The most recent developments and clinical validation are PrPSc-seeded amplification assays [16–18] which increase the level of confidence of the antemortem diagnosis of CJD. Amendment of pre-existing diagnostic criteria by CSF (or other tissue) real time quaking-induced conversion (RT QuIC) has resulted in improved clinical diagnosis and surveillance [19,20].

Despite the availability of highly accurate diagnostic biomarkers, an unmet need in the field is the availability and validation of biomarker tools that might identify people at risk for sporadic prion diseases or predict clinical onset in individuals with *PRNP* mutations. Such markers, as well as dynamic markers that may function as secondary endpoints in clinical trials, are the focus of the current research.

## Disease-related markers and tests in sporadic Creutzfeldt-Jakob disease

In general, there are two classes of protein biomarkers to be considered in neurodegenerative diseases: those related to abnormal concentrations of specific proteins and those related to abnormal protein characteristics. Another consideration should be made with respect to the biomatrix used as a biomarker.

### Quantitative biomarker

In the CSF of patients with prion diseases, abnormal concentrations of brain-derived proteins, such as 14-3-3 protein, tau protein, NfL (Neurofilament light chain), alpha- and beta-synuclein, GFAP, Aβ40/42 and many others are altered, probably because of the general process of rapid neurodegeneration [8]. Novel techniques facilitate the detection and measurement of some of these biomarkers, such as NfL or Tau, in blood plasma or serum [1] to indicate general neuronal damage in prion diseases. Other markers may indicate synaptic damage (beta-synuclein) or glial activation (GFAP). In particular, total Tau and NfL have been shown to be potential multivalent biomarkers in CJD when measured in the CSF or blood plasma (Table 1) [21–23]. Although these markers, and others, are not specific for prion disease, highly increased levels may differentiate between CJD and other neurodegenerative diseases. In this context, NfL has shown a high sensitivity, whereas Tau seems to have better specificity for CJD [21]. These markers show a good correlation between plasma (or serum) and CSF levels in CJD [21], facilitating their application as minimal-invasive markers of disease onset in genetic prion diseases, as well as potential prognostic markers, and markers of disease activity in clinical trials [21–24].

**Table 1.** Current state of biomarkers for prion diseases.

Biomarker	At risk biomarker	diagnostic marker (clinical disease)	prognostic marker (disease progression)	dynamic marker (disease stage/activity)
**Biochemical markers**				
PrPSc CSF	+	+	?	?
PrPSc peripheral fluids	+	+	?	?
14-3-3 CSF	-	+	-	+
Tau CSF	-	+	+	+
Tau Plasma	-	+	+	+
NfL CSF	-	(+)	(+)	(+)
NfL plasma	-	(+)	(+)	(+)
**Other**				
PSWCs in EEG	-	+	-	+
high signal in MRI in FLAIR and/or DWI	(+)	+	-*	(+)

‘+’: Association and/or applicability was shown in significant studies; “-“: Association was not shown in significant studies; ?: no significant data available; ‘()’: In brackets when data is ambiguous or only based on case reports.

*In combination with analysis of the Codon 129 *PRNP* genotype, MRI lesion patterns may contribute to an antemortem disease subtyping and by this, function as a prognostic marker.

### Aggregation assays

Other biomarkers are related to protein characteristics. PMCA (Protein Misfolding Cyclic Amplification) was the developed first and was followed by RT QuIC (Real Time Quaking induced Conversion). The principle of both techniques is the conversion of recombinant prion protein by its interaction with abnormal aggregates of the prion protein (PrPSc seeds). After incubation and direct interaction between the recombinant protein and the seeds, new aggregates were fragmented by sonication (PMCA) or high-frequency shaking (RT QuIC), providing new seeds for the reaction. After repeated cycles of aggregation and shaking, fragmentation, and conversion, the concentration of abnormal PrPSc aggregates is increased and can be detected by fluorescence. The diagnostic accuracy of CSF RT-QuIC in retrospective and prospective studies is very high [8]. Within the last 10 years, a large amount of data is available, and RT-QuIC detection has become part of the clinical routine in specialized CJD laboratories worldwide. The test has been developed for CSF, but also for olfactory mucosa and tear fluids [25].

### The role of brain imaging

Brain imaging, especially MRI, is a crucial part of the diagnostic work-up for prion diseases. Besides high accuracy in the differential diagnosis of CJD, typical MRI lesion patterns of restricted diffusion [8] may contribute to antemortem disease subtyping (with relevance to prognosis) in combination with the analysis of the Codon 129 PRNP genotype [26]. Several case reports have even detected abnormal MRI findings preceding the disease onset of CJD for up to two years [27]. In this context, the potential of MRI as an early clinical or even-preclinical marker of CJD maybe even superior to known fluid biomarkers, which were shown to increase not earlier than close to clinical onset [28]. However, MR scans are laborious and more difficult to perform than simple blood-uptakes, for example. In some genetic prion diseases like GSS and FFI, MRI does not show specific abnormalities in most cases [29].

Another field of emerging techniques is the Positron Emission Tomography (PET). Changes in brain metabolism might help in the differential diagnosis of neurodegenerative disorders by studying disease-specific metabolic networks in individual patients [30].

### Novel developments

Using sensitive techniques such as RT QuIC or PMCA, abnormal PrPSc has been detected in various biofluid island tissues in humans affected by prion disease). PrPSc is not only restricted to the brain and spinal cord but is also found in the olfactory mucosa, lungs, spleen, liver, digestive system, and skin [31,32]. It spreads throughout the body and can therefore be detected in peripheral fluids, which might be more accessible than CSF. There are several advantages, such as less invasiveness and the potential for longitudinal sampling. The idea of developing a blood-based test has been around for many years, but also for urine testing. Another approach to consider might be saliva, sputum, or tear fluid. The latter are easy to collect using a strip of paper, and sampling can be performed by a lay person. Initial data revealed the sensitivity of PrPSc detection in tear fluids in sporadic CJD to be 75% and a specificity of 100% [33].

## Biochemical markers in genetic prion diseases

In general, CSF biomarkers in genetic prion diseases follow a pattern similar to that in sporadic diseases; however, sensitivity differs across mutations. From the biomarker perspective, patients with E200K and V210I mutations are very similar to sporadic CJD, but patients with fatal familial insomnia (FFI) and Gerstmann Sträussler Scheinker syndrome (GSSS) are frequently negative in CSF-based tests for 14-3-3 and tau protein, as well as in the PrPSc RT QuIC reaction [34].

Testing peripheral accessible tissue or biofluids such as tear fluids opens new perspectives in the preclinical diagnosis of human prion diseases. Data from a limited number of patients with various mutations in the prion protein gene tested positive in the RT QuIC. To date, data are available for patients with GSS (P102L), FFI, E200K, or insert mutations. Although the test was positive in affected patients, it also revealed positive results in healthy mutation carriers. According to these data, PrPSc RT QuIC can be positive for at least five years before disease onset. Another potential explanation might be the intrinsic instability of the mutated protein, which allows conversion into the pathological form in the RT QuIC reaction. Other longitudinal studies in cohorts of healthy mutation carriers [28,35,36] point in the same direction. CSF PrPSc RT QuIC has tested positive for several years before disease onset. Interestingly, in converters, NfL is one of the parameters that starts to increase around the clinical onset of the disease [28]. The neurofilament light chain (NfL) has been studied extensively for many neurological disorders and has been shown to be a dynamic biomarker that changes with disease progression.

## Summary and perspectives

Various biomarkers have been identified for prion diseases. Depending on context, they provide different types of information. Some biomarkers provide information on the higher risk of disease (at-risk/prodromal biomarkers). Such biomarkers have been identified for synucleinopathies, as positive alpha-synuclein RT QuIC reactions seem to identify persons at risk of developing synucleinopathy [37,38]. In the context of healthy mutation carriers, PrPSc RT QuIC may have a similar function. At the same time, the detection of PrPSc using RT QuIC can be a diagnostic biomarker when applied to clinical situations.

Another class of quantitative biomarkers can act as biomarkers for disease progression. Candidates have been suggested for this, such as NfL in the plasma. CSF tau levels in CSF correlated with disease progression [39]. Longitudinal studies are required to confirm or disprove this concept.

**Figure 1.**
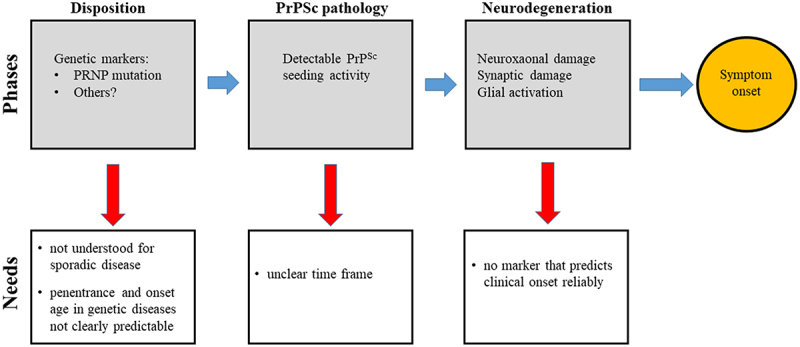
The phases of preclinical prion disease.

In summary, we already developed highly efficient tools for the differential diagnosis of human prion diseases. However, the urgent goal of biomarker research is related to the development and application of therapeutics. This includes characterization of the preclinical phases (Figure 1), refinement of trial endpoint biomarkers, and identification of predictive biomarkers.

## Data Availability

Data sharing is not applicable to this article as no new data were created or analysed for this narrative review article.
